# Knowledge attitude and practice of malignancies among PLWHIV in Nigeria

**DOI:** 10.1186/1750-9378-7-S1-P16

**Published:** 2012-04-19

**Authors:** Clement Adebamowo, Elima Jedy-Agba, Emmanuel Oga, Susan Yilme, William Blattner

**Affiliations:** 1 Office of Strategic Information, Research and Training, Institute of Human Virology, Abuja, Nigeria; 2 Department of Epidemiology, Institute of Human Virology, University of Maryland School of Medicine, Baltimore, MD, USA

## Background

HIV+ individuals are at increased risk of cancers [1]. Data suggest active surveillance and screening are required otherwise cancers in this population will present in advanced stages [2]. Early diagnosis depends on increased awareness. Previous studies noted low levels of awareness of cancers in LMIC and there is need to provide contextual, culturally appropriate health education [3]. We elucidate knowledge, practice and attitude (KAP) of PLWHIV in Nigeria to provide foundation for client education.

## Material and methods

Random sample of HIV+ and HIV- persons in Nigeria, were consented, and asked to participate in FGD on AIDS Associated Malignancies. Each FGD consisted of 10 persons, managed by a researcher and a note-taker using a discussion guide. FGD was recorded, transcribed and analyzed.

## Results

Most participants had heard about cancer and considered it a fatal disease, but they had poor knowledge of the causes. *None had heard of any of the common cancers that occur in PLWHIV.* When asked about specific cancer like Kaposi Sarcoma, Lymphoma and Cervical Cancer, only cervical cancer was mentioned and while they know that it occurs in female reproductive tract, they did not associate it with HIV.

Most respondents did not believe that it is possible to have HIV and cancer though some opined that it may be possible since both are caused by viruses.

Most respondents think that cancer is incurable or treatable by traditional means only.

Participants emphasized use of mass media, community engagement, pre-test counseling and confidentiality as issues that need to be attended to in order to have successful screening program.

## Conclusions

This study showed low levels of awareness of cancer among PLWHIV.
